# Relation Between Calcaneal Fat Pad Thickness and Plantar Foot Ulceration in Patients With Type 2 Diabetes Mellitus

**DOI:** 10.1002/jfa2.70166

**Published:** 2026-07-04

**Authors:** Sem J. van den Nieuwenhof, Margot H. M. Heijmans, Jeroen J. van der Reijden, Chantal D. Bakker, Roel H. D. Vaes

**Affiliations:** ^1^ Department of Surgery Máxima Medical Center Veldhoven the Netherlands; ^2^ Department of Radiology Máxima Medical Center Veldhoven the Netherlands; ^3^ Department of Rehabilitation Medicine Máxima Medical Center Veldhoven the Netherlands

**Keywords:** diabetic foot ulcer, fat pad, MRI, type 2 diabetes mellitus

## Abstract

**Introduction:**

Thinning of fat pads may elevate the risk of developing plantar foot ulcers potentially resulting in lower‐extremity amputations. Evaluating whether fat pad thickness is associated with plantar foot ulceration could aid in early risk detection. This study therefore aims to compare fat pad thickness between type 2 diabetes mellitus (T2DM) patients with and without plantar foot ulcers using MRI.

**Methods:**

This retrospective cross‐sectional study included patients diagnosed with T2DM of whom a nonweight bearing MRI of the ankle or foot was available. The thickness of the calcaneal fat pad was measured in the sagittal plane, measuring perpendicularly from the lowest cortex of the calcaneal bone to and including the skin. Calcaneal fat pad thickness was compared between T2DM patients with and without plantar foot ulceration.

**Results:**

In total, 42 patients were included of which 16 in the T2DM group with plantar foot ulceration. The calcaneal fat pad was significantly thinner in T2DM patients with plantar foot ulceration compared to those without plantar foot ulceration (15.7 ± 3.2 mm vs. 18.6 ± 2.8 mm, *p* = 0.004).

**Conclusion:**

This study established an initial association between fat pad thickness and plantar foot ulceration in T2DM patients. Our findings suggest that a reduction in calcaneal fat pad thickness may decrease the shock‐absorbing ability of the plantar fat pads, making the underlying tissue more vulnerable to injury, increasing the risk of developing plantar foot ulcers. The initial association between fat pad thickness and foot ulceration should be further explored to discover its potential in clinical practice.

## 1 Introduction

In 2021, more than 529 million people globally were diagnosed with diabetes mellitus making it one of the leading causes of death and disability worldwide [1]. By 2050, the number of people suffering from this chronic metabolic disease is expected to rise to 1.3 billion. Over 90% of the people with diabetes mellitus can be attributed to type 2 diabetes mellitus (T2DM) [1].

In T2DM, cells become increasingly resistant to insulin, causing glucose to accumulate in the bloodstream, also known as hyperglycemia [2, 3]. Persistent hyperglycemia can result in various complications, including vascular, neuropathic and biomechanical ones [4, 5]. These complications elevate the risk of developing diabetic foot ulcers (DFU). The exact mechanisms leading to these DFU remain inadequately understood. DFU poses a high risk for infection and is responsible for approximately 80% of lower‐extremity amputations among people diagnosed with diabetes [6]. This is mainly due to widespread, irreversible tissue damage and necrosis [6]. Such amputations profoundly impact patients' quality of life and life expectancy [6]. Prevention of DFU and possible amputations could potentially also save significant societal costs [7, 8].

Previous research has linked qualitative and quantitative changes in the plantar fat pad tissue with an increased risk of developing DFU [9, 10]. The plantar fat pads consist primarily of adipose tissue surrounded by a fibrous tissue septum and are located plantar to the calcaneal and metatarsal bones. They serve a weight‐bearing and shock‐absorbing function to reduce potential injury to the body during ambulation. Whether there is an association between fat pad thickness and plantar DFU has not yet been investigated.

Evaluating whether fat pad thickness is associated with plantar DFU may aid in the prevention or early detection of plantar foot ulceration. This study therefore aims to compare the calcaneal fat pad thickness between T2DM patients with and without plantar DFU using magnetic resonance imaging (MRI). We hypothesize that there will be a difference in fat pad thickness with calcaneal fat pads being thinner in T2DM patients with a plantar DFU compared to T2DM patients without a plantar DFU.

## 2 Materials & Methods

This study is a retrospective cross‐sectional study. Approval of the medical ethical committee was requested but formal approval was deemed unnecessary, according to Dutch law (METC Máxima Medical Center). The study procedure was approved by the Institutional Review Board of Máxima Medical Center.

### 2.1 Participants

CTcue, a search engine for electronic patient records (www.ctcue.com), was used to identify and select patients for inclusion in the study. The search was conducted on May 29, 2024 and was limited to patients from Máxima Medical Centre, Veldhoven, the Netherlands, only. The following inclusion and exclusion criteria were applied.

Patients with plantar DFU were included if they had a documented diagnosis of type 2 diabetes mellitus, an appointment coded specifically for the diabetic foot team, a plantar DFU recorded in the documentation of the departments of surgery, radiology, internal medicine, or rehabilitation, and an appointment at radiology indicating a nonweight‐bearing MRI of the foot performed within 6 months before or 6 months after the onset of the plantar DFU. Patients were excluded if they had objected to the use of their data for scientific or educational purposes.

Patients without a plantar DFU were included if they had a documented diagnosis of type 2 diabetes mellitus and an appointment at radiology indicating a nonweight‐bearing MRI of the foot. Patients were excluded if they had objected to the use of their data for scientific or educational purposes, if a plantar DFU or Charcot foot was documented in the medical records, or if they had an appointment coded specifically for the diabetic foot team.

It should be noted that, due to the availability of MRI appointments in CTcue beginning in November 2017, only scans performed between November 2017 and the date of the search were included in this study. In the group with DFU, if more than one eligible MRI scan was available, the scan performed closest to the onset of the DFU was chosen for inclusion. In the group without DFU, the most recent eligible MRI scan was selected.

### 2.2 Data Collection

CTcue was also used to retrospectively collect data. Baseline characteristics included gender, age at the time of MRI, BMI, smoking behavior (former or current smoker), presence of microvascular complications (neuropathy, retinopathy and nephropathy), presence of macrovascular complications (atherosclerosis, ischemic heart disease, stroke and peripheral vascular diseases), presence of comorbidities (hypertension and hyperlipidemia), the highest HbA1c level prior to ulceration or, for the group without ulceration, before the MRI, history of DFU and time since T2DM diagnosis. The highest HbA1c level before the onset of plantar foot ulceration was chosen because an increase in the HbA1c levels correlates with a higher risk of developing diabetic complications [11].

Although the majority of plantar DFU develop near the metatarsal region, the primary outcome measure of this study was the calcaneal fat pad thickness. The calcaneal fat pad is more favorable than the metatarsal fat pad for assessing fat pad thickness for several reasons. First, the calcaneal fat pad can be relatively easily distinguished from other structures in the foot. Second, the calcaneal fat pad has a relatively large volume resulting in more accurate measurements. Last, measurements of the calcaneal fat pads are less prone to potential deformities and migration of the fat pad [10].

MRI was considered the most appropriate technique to assess calcaneal fat pad tissue. Because all MRI examinations were obtained as part of routine clinical care, acquisition parameters varied depending on the clinical indication. Scans were performed on either 1.5T or 3T systems across two locations using indication specific protocols. The contrast between bone, soft tissues and the skin was consistently high across all scans, allowing an adequate and reliable measurement in all cases, as confirmed through visual assessment by the researchers.

**FIGURE 1:**
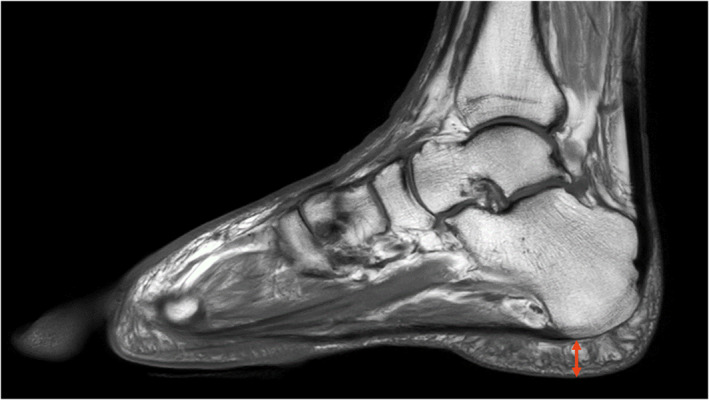
Measurement of calcaneal fat pad thickness in the sagittal plane, measuring perpendicularly from the lowest cortex of the calcaneus to and including the skin.

Enterprise Imaging (AGFA HealthCare Version 8.2.1.060) was used to measure the thickness of the calcaneal fat pad using the available MRI scans. Measurements were performed by two independent researchers (SN and JR). Measurements were conducted in the sagittal plane, measuring perpendicularly from the lowest cortex of the calcaneus to and including the skin (Figure 1). If the measurements of both independent researchers differed less than 2 mm, the average of the two measurements was used for analysis. If the measurements differed more than 2 mm, the patients' MRI was discussed and re‐measured. For all MRIs that were discussed, consensus was reached.

### 2.3 Statistical Analysis

SPSS Version 22 was used to perform the statistical analyses. Baseline characteristics and the outcome measure were compared between T2DM patients with and without plantar DFU. The continuous data were first checked for normality using the Shapiro–Wilk test. If the data were normally distributed, independent samples *t*‐tests were performed. If the data were not normally distributed, the Mann–Whitney *U*‐test was performed. Continuous data were noted as mean ± standard deviation (SD). Categorical data were compared between the two groups using a Chi‐square test. Categorical data were noted as number (%). A *p*‐value < 0.05 was considered statistically significant.

To assess inter‐rater reliability of calcaneal fat pad measurements, a Bland–Altman analysis was performed comparing the two independent assessors. For each measurement pair, the mean value and the difference between assessors were calculated. Limits of agreement were derived as the mean difference ± 1.96 SD. In addition, a linear regression model was fitted with the measurement difference as the dependent variable and the mean measurement as the independent variable to examine the presence of proportional bias.

## 3 Results

The search query resulted in a total of 54 patients. After reviewing the MRI images, 12 patients were excluded since the calcaneal fat pad was not in the field of view. This resulted in a total inclusion of 42 patients of which 16 had a plantar DFU and 26 did not. Dates of the MRI scan ranged from December 2017 until April 2024.

In Table 1, the baseline characteristics of the T2DM patients with and without plantar DFU are shown. There were relatively more males in the group with plantar DFU compared to the group without (81.3% vs. 34.6%, *p* = 0.003). The group with plantar DFU had more microvascular complications than the group without plantar DFU (81.3% vs. 30.8%, *p* = 0.001). The highest recorded HbA1c level before the onset of plantar DFU is higher in the group with plantar DFU compared to the highest recorded HbA1c level before the conducted MRI of the group without plantar DFU (86.8 ± 23.6 vs. 54.2 ± 15.2, *p* < 0.001).

**TABLE 1:** Baseline characteristics of T2DM patients with and without plantar DFU.

	With plantar ulcer (*n* = 16)	Without plantar ulcer (*n* = 26)	*p*‐value
Male, *n* (%)	13 (81.3)	9 (34.6)	0.003^a^
Age, years, mean ± SD	64.1 ± 12.4	63.3 ± 11.7	0.839
BMI, kg/m^2^, mean ± SD	29.9 ± 6.2	32.5 ± 5.3	0.209
Smoking, current or history, *n* (%)	9 (56.3)	17 (65.4)	0.554
T2DM duration, years, mean ± SD	9.1 ± 6.3	10.4 ± 8.7	0.928
Microvascular complications, *n* (%)	13 (81.3)	8 (30.8)	0.001^a^
Macrovascular complications, *n* (%)	9 (56.3)	7 (26.9)	0.057
Comorbidities, *n* (%)	15 (93.8)	20 (76.9)	0.155
Previous diabetic foot ulcer, *n* (%)	8 (50.0)	—	—
Highest HbA1c^b^, mmol/mol, mean ± SD	86.8 ± 23.6	54.2 ± 15.2	< 0.001^a^

*Note:* Age, BMI, T2DM duration and HbA1c are presented as mean ± SD. All other data are presented as raw numbers and percentages.

Abbreviations: BMI: body mass index; HbA1c: hemoglobin A1c; T2DM: type 2 diabetes mellitus.

^a^ *p* < 0.05, which is considered statistically significant.

^b^ With plantar ulcer: 5/16 missing, without plantar ulcer: 12/26 missing.

**FIGURE 2:**
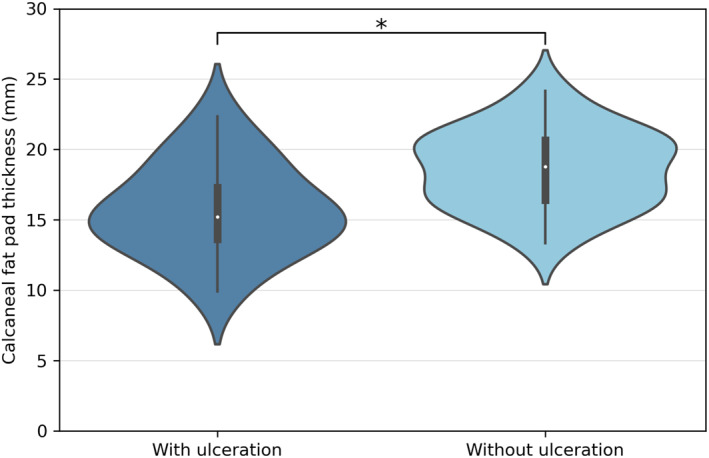
Violin plot showing the calcaneal fat pad thickness of the T2DM group with ulceration (dark blue) and without ulceration (light blue). White dot representing the median, thick black bar representing the interquartile range, and thin black bar representing the data distribution. *p < 0.05.

The thickness of the calcaneal fat pad in T2DM patients with plantar foot ulceration was significantly thinner compared to those without plantar foot ulceration (*p* = 0.004, Figure 2). The mean thickness in the group with plantar foot ulceration was 15.7 ± 3.2 mm, and the mean thickness in the group without plantar foot ulceration was 18.6 ± 2.8 mm.

Since gender differed between the two groups, a logistic regression analysis was performed to examine the association between fat pad thickness, gender and the presence of an ulcer. Increased fat pad thickness was significantly associated with lower odds of DFU presence (OR = 0.693, 95% CI 0.522–0.919, *p* = 0.011), indicating that each mm increase in fat pad thickness reduced the odds of having a DFU by approximately 31%. Gender was also a significant predictor with females showing substantially lower odds of DFU presence compared to males (OR = 0.095, 95% CI 0.017–0.533, *p* = 0.007).

### 3.1 Inter‐Rater Reliability

The Bland–Altman analysis indicated strong inter‐rater agreement with a small mean difference (0.39 mm) and narrow 95% limits of agreement (2.01 mm to −1.23 mm), indicating minimal systematic disagreement. The corresponding Bland–Altman plot is presented in Figure S1. Linear regression showed no evidence of proportional bias between the two raters (*B* = 0.024, SE = 0.043, *p* = 0.576). The intercept did not differ significantly from zero (*B* = −0.036, SE = 0.771, *p* = 0.963), indicating that measurement differences remained consistent across the measurement range.

## 4 Discussion

The increasing prevalence of T2DM has drawn attention to one of its most severe complications: DFU. These ulcerations not only pose a high risk for infection, with dramatic consequences for mobility, but also carry a significant risk for lower extremity amputations [6]. The psychological, social and economic impacts of such amputation are profound, affecting not only the individual but also their families and the healthcare system at large [7, 8]. Understanding the factors associated to the development of DFU and their progression to amputation is crucial for implementing effective preventive and therapeutic measures.

Although the risk of developing a DFU is multifaceted and not fully understood, there are increasing indications suggesting that fat pad thickness plays a role in the risk for developing plantar DFU [9, 10]. This study therefore aimed to investigate whether a possible association exists between the calcaneal fat pad thickness and the presence of plantar DFU in patients with T2DM using MRI. Our results demonstrate that T2DM patients with plantar DFU have thinner calcaneal fat pads compared to diabetics without plantar DFU. These findings suggest an initial possible association in which changes in fat pad thickness may be linked to an increased risk of developing DFU. This may be explained by the fat pads serving a weight‐bearing and shock‐absorbing function. Previous research has shown that a reduction in plantar fat pad thickness likely increases the plantar peak pressure during ambulation, making the skin more vulnerable to injury [10].

The initial association between calcaneal fat pad thickness and plantar foot ulceration in T2DM patients suggests the importance of a renewed approach to plantar foot screening. Foot screening is considered the first line of defense in preventative care in diabetics and is typically performed by regulated healthcare professionals such as podiatrists. Up to now, attention is especially paid to foot morphology, neuropathy, vascular perfusion and skin changes. One might infer that special attention should also be paid to soft tissue changes and loss of plantar fat pad as a potential risk factor for plantar DFU. First, further research is required to determine whether a causal relationship exists between fat pad characteristics and the development of DFU. In addition, continued exploration is needed to determine how plantar fat should be measured and what cut‐off values should be considered for increased risks. Patients with diminished fat pad thickness may eventually receive more frequent preventative screening from a podiatrist or pedicurist to monitor their risk of developing plantar DFU. Also, prescription of offloading appliances can be considered at a low threshold. This way, bothersome complications and the concomitant health care burden may be minimized.

Interestingly, there were several differences between both groups, which could have contributed to the results obtained in this study. First, there were relatively more males in the group with plantar foot ulceration compared to the group without (81.3% vs. 34.6%, *p* = 0.003). Given this substantial difference in sex distribution between groups, gender was included in the multivariable logistic regression model to account for potential confounding. After adjustment for gender, fat pad thickness remained significantly associated with DFU presence, demonstrating an independent effect beyond the influence of sex. Second, there were significantly more patients with microvascular complications in the group with plantar foot ulceration compared to the group without (81.3% vs. 30.8%, *p* = 0.001). Microvascular complications could adversely impact the quantitative plantar fat pad composition. Neuropathy, another consequence of microvascular disease, is likely to result in a loss of pain perception along with the deterioration of intrinsic muscles. The latter can lead to changes in pressure distribution and the migration and thinning of the plantar fat pads [9]. Microvascular complications may therefore be considered a confounder.

Patients with T2DM and plantar foot ulceration demonstrated higher HbA1c values compared with those without ulceration (86.8 ± 23.6 vs. 54.2 ± 15.2, *p* < 0.001). These findings should be interpreted with caution due to the substantial number of missing HbA1c measurements (see Table 1). An HbA1c level ≥ 48 mmol/mol is indicative of diabetes mellitus, and poorer glycemic control, as demonstrated by a further rise in HbA1c levels, is associated with an increased risk of diabetic complications (11, 12). Such differences may partly account for the observed variation in microvascular outcomes. Chronic hyperglycemia promotes the formation of advanced glycation end products (AGEs), which alter extracellular matrix properties and increase tissue stiffness. AGEs have been shown to impair collagen organization and viscoelastic behavior across several tissues [12, 13]. Moreover, AGE‐related changes in adipose extracellular matrix may influence adipocyte function and affect fat pad structure [14]. These mechanisms may be linked to our observations, hypothetically resulting in an apparent reduction in thickness due to degradation of the fat pad tissue structure.

There were a number of limitations to this study. First, the primary reason for patients with plantar foot ulceration to receive an MRI of the ankle or foot is the suspicion of osteomyelitis. Osteomyelitis is an infection and inflammation of the bone that can compromise the overlying plantar fat pads [15, 16]. Theoretically, bias may have occurred by unrecognized osteomyelitis causing fat pad disruption. To address this, the MRI indications for patients with plantar foot ulceration were reviewed. The majority of these patients were suspected of having osteomyelitis in the forefoot. Since the fat pad thickness at the calcaneal bone was measured, it is unlikely that this factor biased the results. Nevertheless, this referral pattern limits the generalizability of our findings to broader diabetic populations, as MRI‐indicated patients may represent a subgroup with more complex pathology. Secondly, the underlying reason for the MRI in the group without foot ulceration was not known during data collection. To mitigate this limitation, patients with MRI images showing abnormalities near the calcaneal fat pad due to a fracture or edema were excluded. However, the remaining patients may still have had foot or ankle conditions that differ systematically from the group with DFU. This uncertainty reduces comparability between groups and should be considered when interpreting the observed associations. Third, because imaging may have been performed after DFU onset, reverse causation cannot be excluded. Fat‐pad thinning could therefore represent a consequence of DFU or related pathology rather than a pre‐existing risk factor. Fourth, the absence of weight‐bearing imaging limits the biomechanical interpretation of our findings, given the load‐dependent nature of plantar soft‐tissue behavior. Lastly, both assessors in this study were nonblinded for the presence of DFU in all measurements. By having two separate individuals measuring the calcaneal pad fat, the study aimed to alleviate this limitation and increase the reliability of the measurements.

Future studies should consider a prospective, double‐blinded approach in a larger population in which T2DM patients receive a MRI of the ankle or foot immediately after developing a DFU regardless of whether there is a suspicion of osteomyelitis. The control group should consist of T2DM patients matched to the T2DM patients with DFU. This approach avoids differences in baseline characteristics and will therefore make the observed results more reliable.

## 5 Conclusion

This study showed that the calcaneal fat pad of T2DM patients with plantar foot ulceration was thinner compared to T2DM patients without plantar foot ulceration. A thinner plantar fat pad is likely to result in lowering the shock‐absorbing ability, posing a higher risk of developing plantar DFU. Practical implications may include structural fat pad thickness measurements in patients with T2DM and the prescription of offloading appliances to manifest insufficient fat pad thickness. This way, troublesome complications and the concomitant health care burden can be prevented. However, due to the cross‐sectional nature of our data, we cannot establish a causal relationship between fat pad thickness and ulceration. The initial association between fat pad thickness and foot ulceration should be further explored to discover its potential in clinical practice.

## Author Contributions

**Sem J. van den Nieuwenhof:** conceptualization, data curation (equal), formal analysis (equal), investigation, methodology (equal), validation, visualization, writing – original draft (supporting). **Margot H. M. Heijmans:** data curation (equal), formal analysis (equal), methodology (equal), project administration, writing – original draft (lead). **Jeroen J. van der Reijden:** investigation (equal). **Chantal D. Bakker:** conceptualization, methodology (equal), validation (equal), supervision (equal), writing – review and editing (equal). **Roel H. D. Vaes:** conceptualization, project administration (equal), validation (equal), supervision (equal), writing – review and editing (equal).

## Funding

The authors have nothing to report.

## Ethics Statement

Approval of the medical ethical committee was requested but formal approval was deemed unnecessary, according to Dutch law (METC Máxima Medical Center). The study procedure was approved by the Institutional Review Board of Máxima Medical Center.

## Consent

The authors have nothing to report.

## Conflicts of Interest

The authors declare no conflicts of interest.

## Supporting information


**Figure S1:** Bland–Altman plot showing agreement between Rater 1 and Rater 2 for calcaneal fat pad measurements. The x‑axis shows the mean of both raters, and the y‑axis shows their measurement difference. The red horizontal line indicates the mean difference, and the green lines represent the 95% limits of agreement (mean difference ± 1.96 SD).

## Data Availability

The data that support the findings of this study are available from the corresponding author upon reasonable request.
