# Supplementary material for: Relation Between Calcaneal Fat Pad Thickness and Plantar Foot Ulceration in Patients With Type 2 Diabetes Mellitus
Source: J Foot Ankle Res. 2026 Jul 4;19(3):e70166. doi: 10.1002/jfa2.70166 (PMC13332807; doi:10.1002/jfa2.70166)
Supplement: Supplementary file 1 — Figure S1: Bland–Altman plot showing agreement between Rater 1 and Rater 2 for calcaneal fat pad measurements. The x‑axis shows the mean of both raters, and the y‑axis shows their measurement difference. The red horizontal line indicates the mean difference, and the green lines represent the 95% limits of agreement (mean difference ± 1.96 SD). [file JFA2-19-e70166-s001.docx]

Supplementary Figure 1: Bland–Altman plot showing agreement between Rater 1 and Rater 2 for calcaneal fat pad measurements. The x‑axis shows the mean of both raters, and the y‑axis shows their measurement difference. The red horizontal line indicates the mean difference, and the green lines represent the 95% limits of agreement (mean difference ± 1.96 SD).
